# Radiographic Outcomes of Thoracolumbar AOSpine A3 and A4 Fractures Treated With External Bracing

**DOI:** 10.7759/cureus.22490

**Published:** 2022-02-22

**Authors:** Paul S Page, Vikas K Parmar, Evalina Bond, Darnell T Josiah

**Affiliations:** 1 Neurosurgery, University of Wisconsin–Madison, Madison, USA; 2 Neurological Surgery, Cleveland Clinic Foundation, Cleveland, USA

**Keywords:** lumbar burst fractures, spine orthosis, lumbar spine surgeries, l1 burst fracture, spine trauma and disease

## Abstract

Background

The treatment of AOSpine A3 and A4 fractures is controversial with no consensus regarding their management in the absence of neurologic deficits. While conservative management with spinal orthosis is a reasonable treatment option, it is believed to run the risk of progressive segmental kyphosis.

Methodology

A retrospective chart review was conducted of all patients undergoing treatment for thoracolumbar burst fractures from T11 to L2. Patients treated with conservative management with lumbar orthosis were included. Upright radiographs at the time of presentation and the one-year follow-up were compared.

Results

In total, 112 patients were evaluated as being treated with thoracolumbar orthosis. Of these, 61 patients presented with A3 fractures compared with 51 who presented with A4 fractures. Of these, two patients in each group failed conservative management and required surgical intervention. At the one-year follow-up, A3 fractures demonstrated an average change in Cobb angle of 4.1 degrees compared with 6.1 degrees in A4 fractures (p = 0.021). In addition, A4 fractures demonstrated a significantly worse kyphotic angle and Gardner angle at the one-year follow-up (p = 0.05 and p = 0.026, respectively).

Conclusions

A3 and A4 fractures can be safely treated with orthosis with overall low rates for failure; however, A4 fractures result in significantly worse segmental kyphosis at the one-year follow-up.

## Introduction

Burst fractures account for approximately 10-20% of all spine fractures, with approximately two-thirds of these fractures occurring at the thoracolumbar junction [1,2]. The presence of burst fractures at this location is largely secondary to the unique biomechanical stress of the juxtaposing static kyphotic thoracic spine and the dynamic and lordotic lumbar spine. Given the variability in the treatment of burst fractures, several classification systems have been developed. While the Thoracolumbar Injury Classification and Severity (TLICS) score is popular given its recommendation for a treatment option, it is often criticized for its ambiguity regarding burst fractures without neurologic deficit. In comparison, the AOSpine Classification system is more descriptive; however, it does not provide treatment recommendations for surgical versus conservative management [3,4]. In this study, we evaluate AOSpine A3 and A4 burst fractures without neurologic deficits treated with an orthosis. Specifically, we evaluate radiographic segmental kyphosis following treatment of A3 and A4 fractures with external bracing.

## Materials and methods

**Figure 1:**
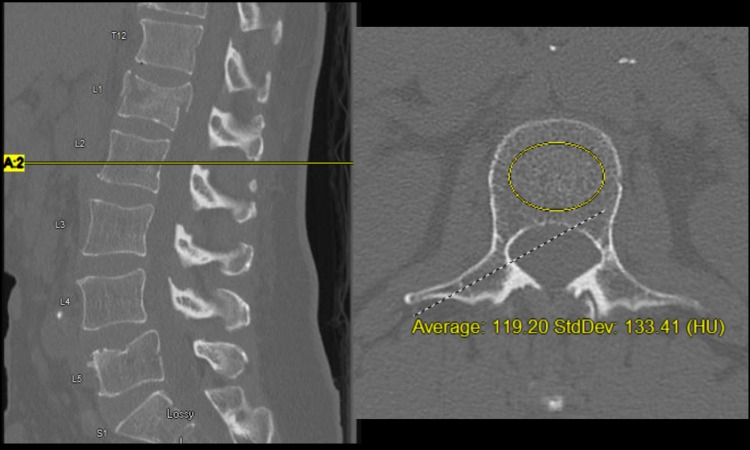
Calculation of the average and standard deviation of Hounsfield Units in the case of a 58-year-old male with an acute L1 burst fracture following a motor vehicle accident.

A retrospective review was conducted evaluating all patients presenting with spine fractures from 2010 to 2017. Inclusion criteria were limited to adults with acute, traumatic burst fractures of the thoracolumbar levels T11-L2. Acute trauma was defined as an injury occurring within three weeks of presentation. Burst fractures were identified as fractures involving the anterior and middle columns with retropulsion of posterior wall bone fragments into the spinal canal. We excluded patients who did not present for follow-up, patients with chronic burst fractures, with the absence of neurologic compromise, with nontraumatic vertebral body collapse (e.g., tumor, tuberculosis), with severe traumatic brain injury, and those with serious injuries associated with other major organs. Total spine computed tomography (CT) scans were obtained for all patients at presentation to the emergency department (ED). Braces selected included either Jewett brace or thoracolumbosacral orthosis (TLSO) bracing. Upright anteroposterior (AP) and lateral plain radiographs were obtained on all patients before discharge from their initial hospital stay and at each follow-up appointment. Final follow-up was defined as radiographs obtained one year from the initial injury. Patients were categorized into A3 and A4 burst fractures based upon the AOSpine Classification system. Data collected from charts included demographic information, comorbidities, level of injury, presence of neurologic deficit at presentation, and imaging characteristics measured from initial and follow-up imaging. Imaging characteristics were obtained including kyphotic angle (KA), Gardner angle (GA), and Cobb angle (CA). A McKesson Picture Archiving and Communications System (McKesson, San Francisco, CA) was used to calculate the average Hounsfield units (HU) by placing an elliptical region of interest confined to the medullary space of the vertebral body at L1 through the middle of the vertebral body on the axial slice (Figure 1). This tool was used as a surrogate marker for the evaluation of bone quality [5]. In cases where the index fracture was located at L1, the L2 vertebral body was utilized.

Categorical variables were assessed using chi-square analysis or analysis of variance. Continuous variables were assessed utilizing the Student’s two-tailed t-test. Change in radiographic measures over time was studied using individual radiographic review at the one-year follow-up across all groups. For all analyses, a significance level of 0.05 was employed. Statistical analysis was performed using Microsoft Excel version 2018.

## Results

In total, 112 patients were evaluated as being treated with thoracolumbar orthosis. Of these, 61 patients presented with A3 fractures and 51 patients with A4 fractures. Baseline patient characteristics between the two groups were similar without any statistically significant difference in age, sex, body mass index, smoking status, fracture level, or bone quality (Table 1). Of these groups, two patients with A3 fractures and two patients with A4 fractures failed conservative management and required surgical intervention. Initial upright X-rays at the time of admission after orthosis fitting demonstrated no significant difference in KA, CA, or GA prior to discharge in A3 or A4 fractures (p > 0.05, Table 2).

**Table 1:** Baseline characteristics of patients presenting with AOSpine A3 and A4 fractures.

Baseline characteristics	A3 fractures (N = 61)	A4 fractures (N = 51)	P-value
Age (years)	54.5 ± 19.5	61.4 ± 19.4	0.064
Sex			
Male (n)	30	23	
Female (n)	31	28	0.71
Body mass index (kg/m^2^)	26.3 ± 6.5	26.6 ± 6.6	0.99
Smoking			
Former (n)	9	5	0.10
Current (n)	14	12	0.94
Fracture level			
T11 (n)	3	2	
T12 (n)	14	12	
L1 (n)	32	28	
L2 (n)	12	9	0.77
Osteopenia (n)	7	12	0.09
Osteoporosis (n)	9	12	0.24
Chronic corticosteroid use (n)	5	4	0.95
Average Hounsfield units ± SD	143 ± 53	122 ± 58	0.054

**Table 2:** Initial radiographic kyphosis in A3 compared to A4 fractures at the time of presentation.

Fracture classification	Initial kyphotic angle	Initial Cobb angle	Initial Gardner angle
A3 fractures (average degree ± SD)	14.78° ± 5.94°	13.03° ± 7.92°	16.83° ± 6.53°
A4 fractures (average degree ± SD)	15.38° ± 7.30°	12.33° ± 7.80°	14.49° ± 8.59°
P-value	0.65	0.65	0.12

Final upright X-rays at one year were then compared to initial upright X-rays at presentation. At the one-year follow-up, A3 fractures demonstrated an average increase in KA of 3.122 ± 2.739 degrees compared with 4.220 ± 2.852 degrees in A4 fractures (p = 0.045, Table 3). In regards to change in CA, A4 fractures demonstrated 6.095 ± 4.789 degrees of progressive kyphosis compared with only 4.089 ± 3.956 degrees in A3 fractures (p = 0.021). This trend was also seen in the change in GA showing a 6.055 ± 4.212 versus 4.416 ± 3.052 change in A4 fractures compared to A3 fractures, respectively (p = 0.026). Additionally, a linear regression analysis was conducted comparing CT HU at the time of presentation compared to change in kyphosis as a surrogate for induvial bone quality. The linear regression analysis did not demonstrate any correlation with HU and radiographic kyphosis with an R2 value of 0.012, 0.003, and 0.158 regarding the change in KA, CA, and GA, respectively.

**Table 3:** Change in radiographic kyphosis in A3 compared to A4 fractures at the one-year follow-up.

Fracture classification	Kyphotic angle	Cobb angle	Gardner angle
A3 fractures (average change in degree ± SD)	3.122° ± 2.739°	4.089° ± 3.956°	4.416° ± 3.052°
A4 fractures (average change in degree ± SD)	4.220° ± 2.852°	6.095° ± 4.789°	6.055° ± 4.212°
P-value	0.045	0.021	0.026

## Discussion

The management of thoracolumbar burst fractures is controversial, with management ranging from no orthosis to multilevel fusion. Due to the large heterogeneity in these fractures and treatment options, several classification systems have been developed to evaluate these fractures. Specifically, the AOSpine Classification was created in 1994 and further characterizes these fractures into complete (A4) and incomplete (A4) burst fractures to better describe the structural integrity of the anterior column. In general, AOSpine Classification is more descriptive; however, it is less frequently utilized in the clinical setting because it does not provide a treatment recommendation. In comparison, while the TLICS system provides this clinical recommendation, the heterogeneity of burst fractures is not well represented. By further subclassifying these fractures and comparing their radiographic outcomes, we aim to provide information to providers regarding expected posttreatment outcomes with external bracing. Given the complicated nature of treatment decisions, such as patient age, pre-presentation spinal alignment, and fragility, this provides further information to providers for making the optimal patient-level decisions [6,7].

In the setting of burst fractures, progressive segmental kyphosis is of particular interest because it can result in worsening pain and associated deformity. Despite this correlation, the correlation between functional outcomes and radiographic kyphosis has been unclearly defined in the literature. In one paper by Cantor et al. (1993), 18 neurologically intact patients were treated with bracing and early ambulation. At the final follow-up, patients were reported to have a good functional recovery as there was no delayed neurologic function, and bed rest was not required [8]. In another retrospective review by Mumford et al. (1988), 41 patients who presented with thoracolumbar burst fractures were evaluated. At the two-year follow-up, 49% had an excellent outcome compared to 22% who had a fair outcome and 12% who had a poor outcome [9]. Additionally, in 2014, Bailey et al. published a randomized controlled trial evaluating patients with A3 burst fractures randomized to orthosis versus no orthosis. In their series, 47 patients were randomized to TLSO placement and 49 received no orthosis. At three months post-injury, patients were found to have no significant change in their Roland Morris Disability Questionnaire score, 6.8 versus 7.7, and a change in kyphosis of six degrees in both groups, regardless of treatment [10]. In our study, external bracing of A4 fractures demonstrated sufficiently worse KA, CA, and GA when compared to A3 fractures.

In addition to fracture classification, osteoporosis has been associated with progressive kyphosis. Given the dependence of the fractured anterior column to provide support, bone quality may be an important variable in the prediction of progressive kyphosis in the setting of burst fractures. A recent study by Seo et al. (2019) evaluated radiographic outcomes in 98 patients following multilevel fusion for thoracolumbar burst fractures. In their series, 43 patients were found to have unfavorable radiographic outcomes, as defined by either instrumentation failure or abnormal thoracolumbar alignment [11]. On multivariate logistic regression testing, it was shown that osteoporosis was a strong predictor of this outcome (p = 0.049). In our study, HU and a known history of osteoporosis or osteopenia were utilized as surrogate markers of bone quality [5,12]. While dual-energy X-ray absorptiometry (DEXA) scan is the gold standard for evaluating bone quality, the use of HU on CT has been well documented to correlate with DEXA scans and is universally available as most burst fractures are detected initially on CT imaging. In our series, HU was not found to correlate with either A3 or A4 fractures as well as with progressive kyphosis when treated with external bracing.

Despite a large number of patients in our study, significant limitations exist. While the AOSpine classification is more descriptive than the Denis system or TLICS, much heterogeneity exists regarding the degree of comminution and the apposition of fragments. Future studies can evaluate these fractures to see if the McCormick Load-Sharing Classification may be a useful way to predict progressive kyphosis when considering external bracing [13]. Additionally, our study does not include patient-reported outcome measures or clinical outcomes, which is a major limitation. Furthermore, because bracing was done in the outpatient setting, compliance was difficult to monitor in this population. We recommend future studies to evaluate clinical outcome measures in comparison to change in kyphosis to identify what extent of kyphosis results in clinically significant consequences.

## Conclusions

Treatment of thoracolumbar burst fractures with an external orthosis is a safe option in the setting of A3 and A4 fractures; however, A4 fractures result in statistically significantly worsening segmental kyphosis.
